# Biochemical Mapping of *Pyrodinium bahamense* Unveils Molecular Underpinnings behind Organismal Processes

**DOI:** 10.3390/ijms222413332

**Published:** 2021-12-11

**Authors:** Bryan John J. Subong, Zabrina Bernice L. Malto, Arturo O. Lluisma, Rhodora V. Azanza, Lilibeth A. Salvador-Reyes

**Affiliations:** Marine Science Institute, College of Science, University of the Philippines Diliman, P. Velasquez St., UP Diliman, Quezon City 1101, Philippines; bjsubong@msi.upd.edu.ph (B.J.J.S.); zlmalto@up.edu.ph (Z.B.L.M.); aolluisma@up.edu.ph (A.O.L.); rvazanza@up.edu.ph (R.V.A.)

**Keywords:** *Pyrodinium bahamense*, proteomics, biochemical pathways, harmful algal bloom, paralytic shellfish toxins, biomolecules

## Abstract

Proteins, lipids, and carbohydrates from the harmful algal bloom (HAB)-causing organism *Pyrodinium bahamense* were characterized to obtain insights into the biochemical processes in this environmentally relevant dinoflagellate. Shotgun proteomics using label-free quantitation followed by proteome mapping using the *P. bahamense* transcriptome and translated protein databases of *Marinovum algicola*, *Alexandrium* sp., *Cylindrospermopsis raciborskii*, and *Symbiodinium kawagutii* for annotation enabled the characterization of the proteins in *P. bahamense*. The highest number of annotated hits were obtained from *M. algicola* and highlighted the contribution of microorganisms associated with *P. bahamense*. Proteins involved in dimethylsulfoniopropionate (DMSP) degradation such as propionyl CoA synthethase and acryloyl-CoA reductase were identified, suggesting the DMSP cleavage pathway as the preferred route in this dinoflagellate. Most of the annotated proteins were involved in amino acid biosynthesis and carbohydrate degradation and metabolism, indicating the active roles of these molecules in the vegetative stage of *P. bahamense*. This characterization provides baseline information on the cellular machinery and the molecular basis of the ecophysiology of *P. bahamense*.

## 1. Introduction

*Pyrodinium bahamense*, which was first studied by L. Plate in 1906, is the primary etiologic agent of harmful algal bloom (HAB) occurrences in tropical and subtropical areas, particularly in Southeast Asia [1,2]. *P. bahamense* was first identified as a separate genus from *Gonyaulax* (modern name: *Alexandrium*) in 1906. It was observed that the former has morphological differences from the latter, such as a shorter apical horn and antapical spine and a more anterioposteriolly compressed body [3]. Morphologically, *P. bahamense* cells are subspherically to laterally ellipsoidal with thecal plates and ornamented with an apical projection or node. Further, molecular phylogenetic inference using nuclear-encoded small subunit (SSU) ribosomal RNA genes, large subunit (LSU) rRNA gene, and mitochondrial cytochrome b showed the *P. bahamense* clade under Gonyaulacales [4,5]. 

Toxic blooms of *P. bahamense* became prominent in the 1970s in the Indo-Pacific Ocean and the Pacific coast of Central America. *P. bahamense* has the greatest number of reported cases of human illnesses and fatalities among the paralytic shellfish toxin (PST)-producing dinoflagellates [6]. Toxin production and eco-physiological interactions of *P. bahamense* have been extensively characterized by our laboratory [1,2,7]. The toxin content of *P. bahamense* is location dependent, with Indo-Pacific isolates containing saxitoxin (STX), decarbamoyl saxitoxin (dcSTX), neo-STX, B1, and B2 [7,8]. In contrast, Guatemalan isolates produce gonyautoxin (GTX) 2, GTX3, and GTX4 [9]. The Atlantic strain of *P. bahamense* from the Indian River Lagoon, Florida, USA yielded STX, dcSTX, and B1 [10]. 

Compared to other dinoflagellates, *P. bahamense* is more resistant to germination from the cyst stage; hence, changes in abiotic factors may lead to the possible germination of long-standing cyst densities and cause further HAB formation [11,12]. Factors such as temperature [11], anoxic conditions [13], and potential culture stresses leading to genetic changes [14,15] have been implicated in germination resistance. Structurally, *P. bahamense* has a highly resistant cyst wall that is hard to degrade even after germination [16,17]. These factors, including formation of temporary cysts, resistant thick-walled cyst formation, and germination due to changes in environmental conditions, have been proven vital to HAB dynamics [16,18].

A bacterial theory associated with the toxicity of *P. bahamense* has also been proposed [19], where the symbiotic relationship between prokaryotes and *P. bahamense* is crucial to toxin biosynthesis and other key processes in *P. bahamense* [19,20]. Using 16S rDNA sequence analyses, culturable microbiota associated with *P. bahamense* were mapped onto the phylum Proteobacteria [19]. Several of the identified associated bacteria were capable of producing PSTs. Further, 16s rRNA sequencing-based analysis on the associated bacteria of *P. bahamense* indicated the dominance of potential DMSP-degrading *Roseobacter* sp. under the phylum Proteobacteria, class Alphaproteobacteria in *P. bahamense* from the Philippines [21]. Metagenomic analysis of *P. bahamense* from Malaysia similarly showed the phyla Proteobacteria as the major bacteria associated with *P. bahamense* [22]. These studies highlighted the potential role of the phylum Proteobacteria in *P. bahamense* biology.

Despite the extensive characterization of the ecophysiology of *P. bahamense*, there is still limited information on the biochemistry of this organism. Genome characterization has been challenging for *P. bahamense* and other dinoflagellates due to the large size and unusual genome organization [23,24]. Biological mass spectrometry and transcriptome sequencing have been utilized to characterize the biomolecules and cellular processes in microalgae and dinoflagellates [25,26,27,28]. These provided information on organismal processes such as cell growth [29], toxin biosynthesis [30,31,32], and lipid and carbohydrate production [33,34,35].

Shotgun proteomics using mass spectrometry has revolutionized the global analysis of the complex mixture of proteins from an organism. This approach requires the proteins to be enzymatically digested to smaller peptides that are subsequently sequenced by mass spectrometry [36]. Protein levels can be assessed using chemical tags or label-free quantitation (LFQ). Isobaric tags for relative and absolute quantitation (iTRAQ), Isotope-coded affinity-tag-based protein profiling (ICAT), and stable isotope labeling by amino acids in cell culture (SILAC) are examples of peptide-labeling techniques [32,37,38,39,40]. LFQ, on the other hand, utilizes mass spectral peak intensities or spectral counting to determine the protein abundance, hence, foregoing the need for additional labeling steps or chemical tags [41,42]. 

In this paper, we performed a mass spectrometry- and chromatography-based biochemical mapping of the proteins, lipids, and carbohydrates of *P. bahamense* to characterize the cellular processes and machinery of this HAB-causative organism. The proteome was profiled with shotgun proteomics using label-free quantitation, while lipid and carbohydrates were analyzed using gas chromatography and electrospray ionization mass spectrometry, respectively. 

## 2. Results and Discussion

The average moisture and ash contents of *P. bahamense* biomass were 2.392 ± 0.004% *w*/*w* and 18.47 ± 0.09% *w*/*w*, respectively. Total sugar and fatty acid contents in *P. bahamense* were 20.31 ± 2.60% *w*/*w* and 18.12 ± 3.19% *w*/*w* of the total biomass, respectively. Protein and other components were estimated to be ~40.71% *w*/*w* of the total biomass of *P. bahamense* (Figure S1). Proteins, lipids, and carbohydrates in *P. bahamense* were characterized to aid in the understanding of organismal processes. 

### 2.1. Proteome Analysis of P. bahamense

Shotgun protein analysis of *P. bahamense* for two biological replicates was performed using label-free quantitation through spectral counting. The resulting sequences were analyzed with the NCBInr database using MASCOT v2.5.1 (Matrix Science: Columbus, OH, USA, 2014), which had 90,971,994 sequences and 33,504,913,701 residues available during the query. This generated 11,102 queries that resulted in 627 total protein matches (including non-unique proteins, e.g., isoforms) for the first biological replicate (Supplementary Material Dataset 1). The second biological replicate generated 8236 queries and 592 total protein matches (including non-unique proteins, e.g., isoforms) (Supplementary Material Dataset 1). A total of 110 unique GeneInfo (gi) identifiers were identified for both biological replicates (Figure S2). Additionally, the first and second biological replicate gave 91 and 181 unique gi identifiers, respectively. The false discovery rate (FDR) was <1% (95% confidence interval) (Supplementary Material Dataset 1). Identified proteins were further annotated using UniProtKB (https://www.uniprot.org/; accessed on 1 December 2016) and Gene Ontology (GO) feature of UniProtKB (https://www.uniprot.org/uploadlists/; accessed on 1 December 2016). Identified proteins were 89% bacterial and 11% eukaryotic. From the 201 identified gene accessions derived from NCBInr, 188 were successfully mapped onto 197 UniPROTKB ID. The majority of the protein hits (96%) were traced to *Proteobacteria* (Figure S3), specifically *Marinovum algicola*, previously identified as *Ruegeria atlantica*. 

**Figure 1:**
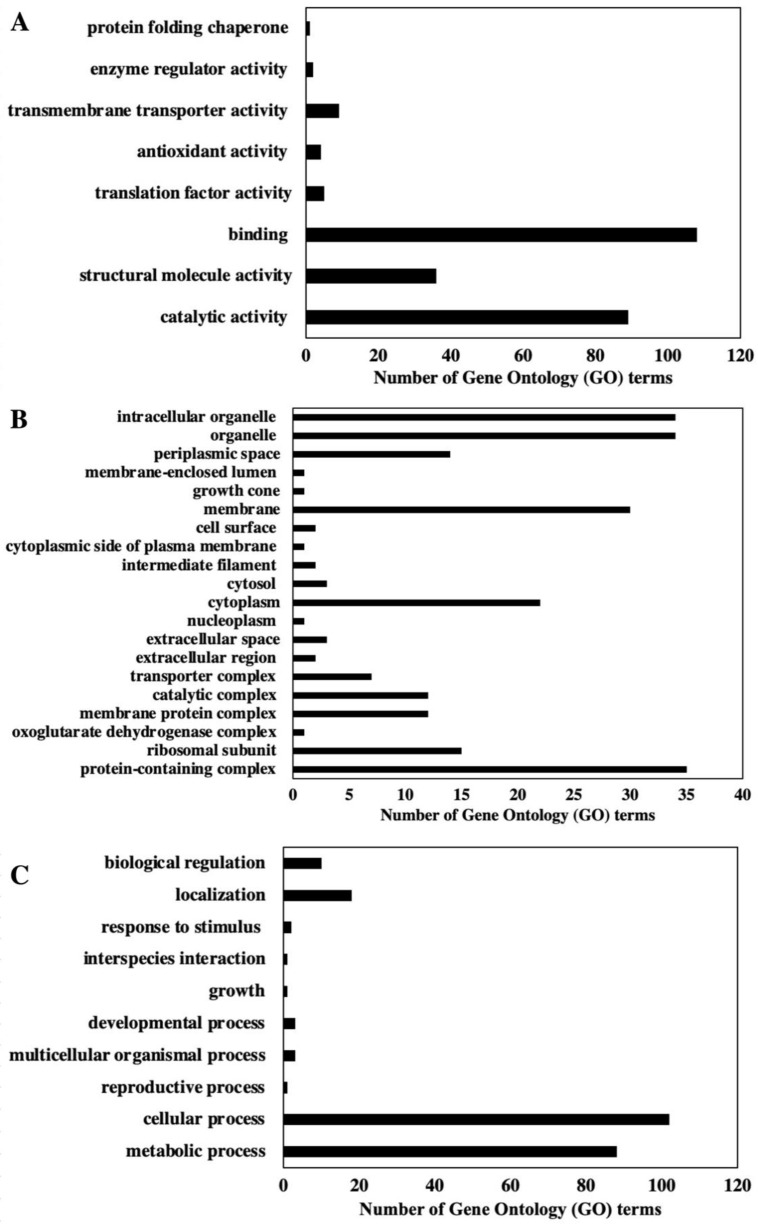
Gene Ontology (GO) terms for the molecular function, cellular components, and biological process based on proteins identified using the NCBInr database search: (**A**) Molecular function GO term distribution highlights binding, structural molecule activity, and catalytic activity as top three annotated GO terms. (**B**) Cellular component GO terms include organelle, intracellular organelle, and protein containing as top three GO term hits. (**C**) Biological process GO terms include cellular process, metabolic process, and localization as the three most associated GO terms with the proteins identified.

**Figure 2:**
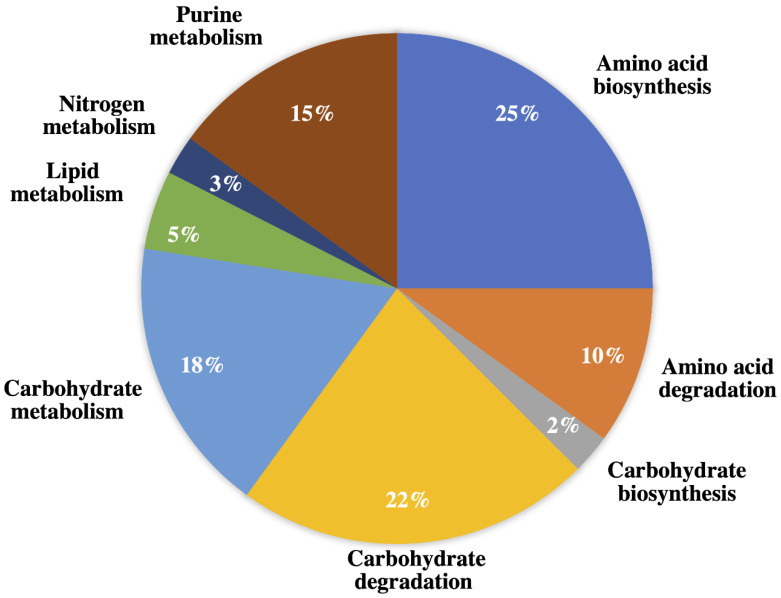
Distribution of proteins in *P. bahamense* based on annotated roles in biological pathways. Using NCBInr database search, the total proteins for two biological replicates were annotated using UniProt (https://www.uniprot.org/). A total of 93.5% of the total proteins identified were successfully mapped onto UniProtID for annotation. Identified proteins are involved in amino acid biosynthesis (25%), carbohydrate degradation (22%), carbohydrate metabolism (18%), purine metabolism (15%), amino acid degradation (10%), lipid metabolism (5%), nitrogen metabolism (3%), and carbohydrate biosynthesis (2%). Proteins from vegetative cells of *P. bahamense* were extracted using TCA precipitation, and shotgun proteome analysis was carried out using label-free quantitation through spectral counting.

Among gene ontology terms for the annotated proteins, 39% of the proteins are involved in molecular function, 28% are cellular components, and 33% of the proteins identified are involved in biological processes (Figure 1 and Figure 2, Figures S3 and S4). In terms of molecular function, binding, catalytic activity, and structural molecule activity are the three dominant functions. Organelle, intracellular organelle, and protein-containing complexes are the three dominant cellular components. Cellular process, metabolic process, and localization are the three major biological processes based on GO term annotation (Figure 1).

**Figure 3:**
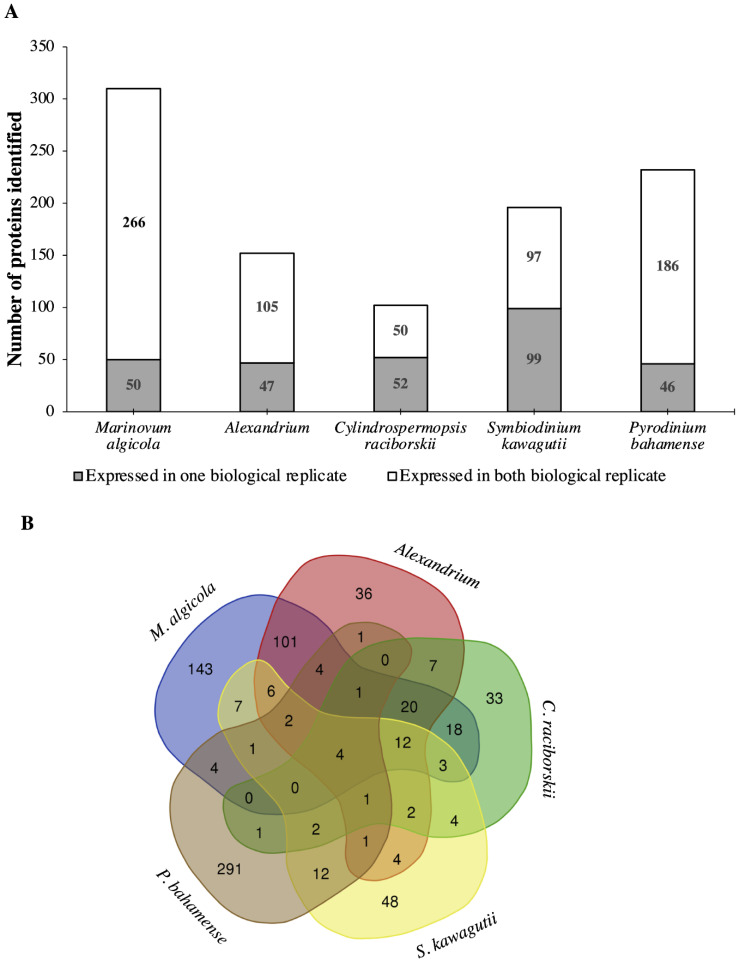
Taxonomic distribution of protein hits from *P. bahamense* and Gene Ontology (GO) distribution across different reference genomes: (**A**) Highest hits were observed using *M. algicola* and *P. bahamense* as reference. Majority of the annotated proteins were detected in the two biological replicates. Several proteins were detected in only one replicate and may indicate possible protein variation due to regulation [45,46] and/or low abundance proteins. (**B**) The highest number of non-overlapping GO identifiers were mapped onto *M. algicola* and *P. bahamense*. Proteins identified were sorted according to their gene ontology (GO) identifiers. Annotation of the GO ID produced the following unique elements or non-redundant GO identifications: *Alexandrium* sp. (36), *C. raciborskii* (33), *M. algicola* (143), *P. bahamense* (291), and *S. kawagutii* (48).

Restricted database search (MaxQuant v1.5.5.1; Max Planck Institute of Biochemistry: Munich, Germany, 2016) using translated protein or transcriptome-dependent protein identification was performed using *Marinovum algicola*, *Alexandrium* sp., *Cylindrospermopsis raciborskii*, *Symbiodinium kawagutii*, and *P. bahamense* as references (Supplementary Material Dataset 2). *M. algicola* was chosen as a reference genome because of the high number of protein hits based on the MASCOT search. The related *Alexandrium* sp., *C. raciborskii*, and *S. kawagutii* (Symka database) were further utilized as references due to the relation between the biochemical machineries of these organisms and *P. bahamense* [43,44]. Finally, an in-house *P. bahamense* transcriptome was used to map the proteome of *P. bahamense*. The number of proteins identified from each reference proteome and transcriptome with corresponding Gene Ontology (GO) distribution is shown in Figure 3a.

The proteins identified across different genomes were annotated using Gene Ontology identification (GO ID) and processed and visualized using REVIGO (http://revigo.irb.hr/; accessed on 30 June 2021) for biological process and molecular function (Figure S5). Annotation of the GO ID produced the following unique elements or non-redundant GO identifications: *Alexandrium* sp. (36), *C. raciborskii* (33), *M. algicola* (143), *P. bahamense* (291), and *S. kawagutii* (48) (Figure 3B). While *M. algicola* had the highest number of annotated hits (Figure 3A), the *P. bahamense* transcriptome provided the greatest unique GO ID, highlighting the importance of transcriptomics of a non-model organism in mapping the proteome. These non-overlapping GO ID often suggest protein and protein functions that are unique to that specific organism [47]. Unique GO identifiers using *P. bahamense* transcript as reference yielded a wide range of processes that include proton transmembrane transport, carbohydrate metabolic process, response to endoplasmic reticulum stress, aromatic compound biosynthesis, sulfur compound biosynthesis, cellular protein and macromolecule process, small molecule process, hexose metabolic process, response to biotic and abiotic stimuli, cell communication, cellular response to organic substances, regulation of responses to stimulus, tetrapyrrole biosynthesis and metabolism, macromolecule localization, defense response, and indole-containing compound metabolic process. Several proteins were expressed in one biological replicate (Figure 3A) and may indicate possible regulation in protein expression, while proteins that were constantly observed in both replicates suggest constitutive expression. Low abundance proteins are also more challenging to detect due to instrument limitation.

#### 2.1.1. Interactions between *P. bahamense* and Associated Microorganisms

An important aspect of the biology of *P. bahamense* is the role of associated microorganisms in various cellular processes. Metagenomics study of *P. bahamense* Malaysian isolate showed that associated bacteria mainly belong to the phylum Proteobacteria, accounting for 69.5% of the total bacterial community, the most dominant class being Alphaproteobacteria (60.5% of all Proteobacteria) [22]. The parallel study on *P. bahamense* Philippine isolate showed *Roseobacter* sp. clade, belonging to the class Alphaproteobacteria, accounting for 68% of the total identified associated intracellular microbiome [21]. 

In this study, the highest number (316) of annotated proteins were matched to *Marinovum algicola*, belonging to the *Roseobacter* clade. The high number of annotated proteins identified from *M. algicola* reference genome supports earlier findings that *Roseobacter* sp. may play a pivotal role in *P. bahamense* biology. These bacterial-origin proteins may be essential for *P. bahamense*, as removal of associated microorganisms by antibiotic treatment significantly affects the survival of *P. bahamense* cultures [48]. 

Based on the sequence, relevant proteins with bacterial origin (*Roseobacter* clade) include amino acid ABC transporter substrate-binding proteins, elongation Tu factor, RHS-repeat-associated core-containing protein, propionyl-CoA synthethase, acryloyl-CoA reductase, and bifunctional folylpolyglutamate synthase/dihydrofolate synthase (Table S2). Amino acid ABC transporter substrate-binding proteins are often used by bacteria to assimilate inorganic nitrogen from the environment to the cell of dinoflagellates [49], and elongation Tu factor is involved in the synthesis of proteins during translation [50], while RHS-repeat-associated core-containing protein has been implicated in toxin production [51].

**Figure 4:**
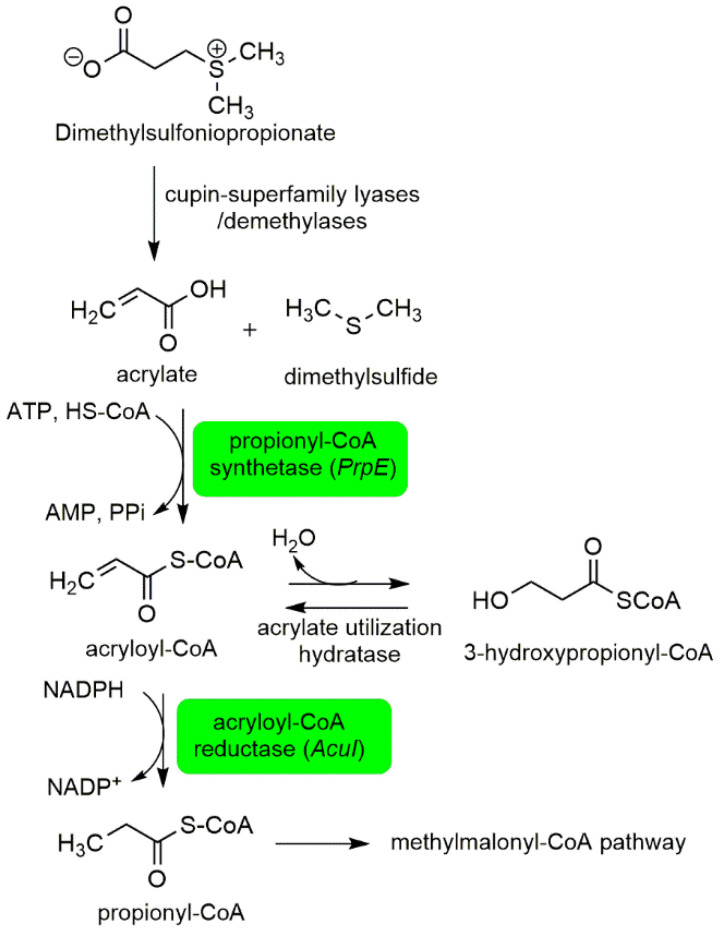
Dimethylsulfoniopropionate degradation through the cleavage pathway. Propionyl-CoA synthetase and acryloyl-CoA reductase (in green) were identified in *P. bahamense* using *Marinovum algicola* reference database. These enzymes are mainly involved in the cleavage pathway for the degradation of dimethylsulfoniopropionate. Propionyl-CoA synthetase catalyzes the conversion of acrylate to acryloyl-CoA. Acryloyl-CoA is then converted to propionyl-CoA through catalysis of acryloyl-CoA reductase. The presence of these proteins in the proteome of *P. bahamense* corroborates the previous reports on the role of associated bacteria in this process. This process is also likely to contribute to the cellular energetics of *P. bahamense* through the generation of propionyl-CoA, an intermediate for the biosynthesis of precursors in the Krebs cycle. Highlighted proteins in green were detected in the present study. Figure was adapted from Bullock et al., 2017 [53].

Associated bacteria in *P. bahamense* were previously proposed to contribute to dimethylsulfoniopropionate (DMSP) degradation [21]. DMSP is a metabolite produced by marine phytoplankton and a major precursor to the climatically important gas dimethylsulfide [52]. Dimethylsulfide (DMS) and methanethiol are volatile organic sulfur compounds that play a major role in the global sulfur cycle [53]. DMS is oxidized in the atmosphere by hydroxyl and nitrate radicals to produce degradation products such as CO_2_, dimethyl sulfoxide, and sulfates [54]. Bacterial DMSP degradation can occur in two pathways—the demethylation and the cleavage pathway [53]. Here, we observed two proteins involved in the DMSP cleavage pathway: propionyl-CoA synthethase and acryloyl-CoA reductase (Figure 4). Propionyl-CoA synthethase catalyzes the conversion of acrylate to acrylol-CoA. Acryloyl-CoA is converted to propionyl-CoA through acryloyl-CoA reductase. Propionyl CoA is among the key building blocks to biosynthetic intermediates in the Krebs cycle, methylmalonyl CoA and succinyl CoA. The DMSP pathway potentially contributes to the cellular energetics of *P. bahamense*. While other proteins implicated in the DMSP-demethylation pathway were not observed in this study, the identification of propionyl-CoA synthethase and acryloyl-CoA reductase corroborates the earlier report on the potential degradation of DMSP in the intracellular region of *P. bahamense* by associated microorganisms. 

A bifunctional folylpolyglutamate synthase/dihydrofolate synthase was also annotated in the *P. bahamense* proteome. It is, however, unusual for eukaryotes to express these proteins since dihydrofolate synthase and folylpolyglutamate synthase are expressed differently. These two proteins are essential for cell proliferation since they are involved in folate biosynthesis and modification. *Plasmodium falciparum* is among the few exceptions in having a single bifunctional protein for folate biosynthesis [55]. *Symbiodinium* *kawagutii* and *Symbiodinium* *minutum* share strong protein homology with *P. falciparum*, especially with proteins involved in parasite–host interactions [39]. The bifunctional folylpolyglutamate synthase/dihydrofolate synthase may be key in understanding the molecular mechanism of *P. bahamense* growth and proliferation. 

#### 2.1.2. Toxin Production 

At the time of collection, subcultured *P. bahamense* had a toxin content of 200–215 fmol STX equiv cell^−1^ (Figure S6). The same strain of *P. bahamense* from the Philippines was reported earlier to have a toxin content peak of 298 fmol STX equiv cell^−1^ observed at the mid-exponential phase and the lowest toxin content at 54 fmol STX equiv cell^−1^ at the death phase [7], with saxitoxin as the major toxin. Malaysian isolates contained 400 fmol STX equiv cell^−1^, with the lowest content at 200 fmol STX equiv cell^−1^ during the stationary phase [8]. 

**Figure 5:**
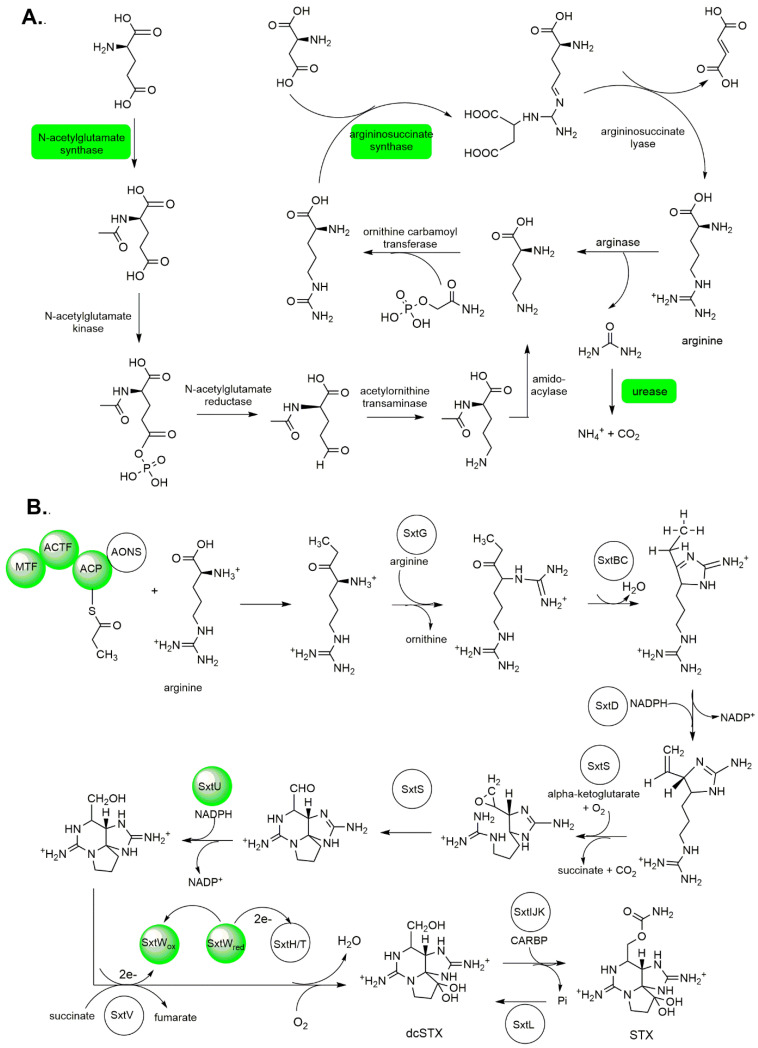
Arginine and paralytic shellfish toxin biosynthesis in *P. bahamense*: (**A**) Proteins involved in arginine biosynthesis such as ornithine decarboxylase, argininosuccinate synthase, ornithine-acyl-ACP acyltransferase, and bifunctional ornithine acetyltransferase/N-acetylglutamate synthase were detected in this study. Nitrogen metabolism in *P. bahamense* was mapped onto urea degradation, where carbon dioxide and ammonia are produced via the urease route. Figure adapted from Bromke et al. 2013 [60]. (**B**) Arginine serves as one of the building blocks for paralytic shellfish toxin biosynthesis. The detected proteins involved in arginine and PST biosynthesis in this study are highlighted in green. Figure adapted from Akbar et al. 2020 [59]. STX, saxitoxin; dcSTX, decarbamoylsaxitoxin.

To understand the toxin production of this organism, we mapped proteins that may be directly and indirectly involved in saxitoxin biosynthesis, arginine biosynthesis, and polyketide synthesis pathway (Figure 5). Despite the five reference genomes utilized for annotation, we only identified 20 proteins that are potentially relevant to organismal toxicity (Tables S2–S4), comparable to previous proteomic studies on other dinoflagellates [32,56].

Some of the putative indirect toxin biosynthesis-related proteins we identified include chlorophyll a/c binding protein and an RHS-associated core domain containing protein (Table S2). Chlorophyll a/c binding protein is an indirect protein involved in saxitoxin biosynthesis and functions as a housekeeping protein in photosynthesis [57]. An RHS-associated core domain containing protein is part of a protein complex implicated in the synthesis of bacterial exotoxins [51].

We mapped the PST biosynthetic enzyme machinery (Table S3) based on the saxitoxin biosynthesis pathway in *C. raciborskii* and omics-driven dinoflagellate toxin biosynthesis pathway elucidation [58,59]. These proteins include adenosylhomocysteinase SAH, methyltransferase, polyketide synthase, acetyl coenzyme A (acetyl-CoA) transferase, histidine kinase, alcohol dehydrogenase, ferredoxin-binding protein, and ferredoxin-nitrite reductase. In the earlier established *C. raciborskii* saxitoxin biosynthesis pathway [58], the initial synthesis step involves S-methyltransferase, adenosylhomocysteinase SAH, and polyketide synthase. Histidine kinase is a putative transcriptional regulator of saxitoxin biosynthesis. Alcohol dehydrogenase catalyzes the reduction of the terminal aldehyde of (8S)-6-amino-9-formyl-1,5,7,10-tetraazatricyclo [6.3.1.0^4,12^]dodeca-4,6-dien-11-iminium to form {2,6-diimino-octahydropyrrolo[1,2-c]purin-4-yl}methanol. Meanwhile, ferredoxin is one of the enzymes involved in the conversion of the pre-saxitoxin intermediate (8S)-9-[(carbamoyloxy)methyl]-1,5,7,10-tetraazatricyclo [6.3.1.0^4,12^]dodec-4-ene-6,11-bis(iminium) to saxitoxin [58,59] (Figure 5B).

Proteins involved in arginine biosynthesis such as ornithine decarboxylase, argininosuccinate synthase, ornithine-acyl-ACP acyltransferase, and bifunctional ornithine acetyltransferase/N-acetylglutamate synthase (Table S4, Figure 5A) were mapped in *P. bahamense*, with *M. algicola* and *Alexandrium* as reference databases. Argininosuccinnate synthase was previously reported as a putative protein involved in saxitoxin biosynthesis [49], and bifunctional ornithine acetyltransferase/N-acetylglutamate synthase has been proposed to have a potential role in toxin biosynthesis based on protein interaction network analysis [31]. Polyketide synthase and type 1 polyketide synthase-related proteins (Table S5) were also identified in *P. bahamense* proteome. These include beta-ketoacyl synthase, which is responsible for one round of chain extension, and beta keto-acyl reductase, dehydratases (acyl-carrier-protein), enoyl reductase, and acyltransferase, which are involved in post-condensation modifications.

Despite this wealth of information, mapping the entire toxin biosynthesis route of *P. bahamense* remains a challenge comparable with previous proteomic studies of toxic dinoflagellates [32]. Factors such as protein expression levels, kinetics, and potential periodicity or biological clock rhythm are some of the possible limiting factors.

#### 2.1.3. Amino Acid Biosynthesis, Degradation, and Nitrogen Metabolism

Proteins involved in amino acid biosynthesis and degradation were mapped in the proteome of *P. bahamense*. These amino acid biosynthesis pathways include the L-ornithine and carbamoyl phosphate pathways producing L-arginine, the S-adenosyl-homocysteine pathway producing L-homocysteine, the 2-oxobutanoate pathway producing L-isoleucine, the 3-methyl-2-oxobutanoate pathway producing L-leucine, the diaminopimelate (DAP) pathway producing L-lysine, and the L-methionine biosynthesis via de novo pathway, with L-aspartate leading to L-homoserine and L-threonine production, 3-phospho-D-glycerate leading to L-serine biosynthesis, the pyruvate pathway producing L-valine), and L-methionine leading to S-adenosyl-L-methionine production (Supplementary Material Dataset 1). These findings are analogous to the observed prevalence of proteins involved in the biosynthesis of methionine, cysteine, ornithine, and arginine in toxic *Alexandrium* species [61].

The mapped specific degradation pathways of amino acids for *P. bahamense* were L-alanine degradation via the dehydrogenase pathway, which produces ammonia and pyruvate and L-lysine degradation via the saccharopine pathway, which produces glutaryl-CoA and L-valine degradation. Among eukaryotes, L-alanine is degraded through the dehydrogenase pathway, where alanine is converted to pyruvate through transamination. This assimilation of L-alanine often serves as an energy source for various organisms through the tricarboxylic acid cycle (TCA) cycle. Moreover, L-lysine degradation through the saccharopine pathway produces glutaryl-CoA. This process involves an atypical transamination reaction in which the α-amino group of lysine is transferred to α-ketoglutarate to form glutamic acid. This pathway is the major lysine degradation pathway for plants, animals, and bacteria [62]. The degradation of L-valine involves the conversion of 3-methyl-2-oxobutanoate to isobutanoyl-CoA. The final product in the L-valine degradation is propionyl-CoA, which then enters the TCA or Krebs cycle. The various degradation pathways mapped in *P. bahamense* suggest that the end products are likely to be involved in nutrient assimilation and serve as an energy source.

Nitrogen metabolism in *P. bahamense* was mapped onto urea degradation, where carbon dioxide and ammonia are produced via the urease route (Figure 5A, Supplementary Material Dataset 1). Proteins detected in *P. bahamense* include assimilatory nitrite reductase large subunit and urease subunit alpha. Assimilatory nitrite reductase large subunit is a protein responsible for nitrate reduction (assimilation), while the urease subunit alpha is involved in the synthesis of urea from carbon dioxide and ammonia.

A recent environmental study showed that urea contributes to more than half of the total nitrogen required by phytoplankton in estuaries and coastal waters. This substantial portion of nitrogen demand affects the proliferation of HAB-causing dinoflagellates [63]. Interestingly, the detected urease subunit alpha sequence in our study was mapped onto *M. algicola*. This may suggest a potential role of associated bacteria for urea production in *P. bahamense*.

#### 2.1.4. Photosynthesis and Bioluminescence

*P. bahamense* contains rubisco (ribulose biphosphate carboxylase, chloroplastic) form II, which is generally a nuclear genome-encoded protein. *P. bahamense* rubisco was identified using the *Symbiodinium* reference genome (Table S6). The presence of rubisco form II among dinoflagellates, particularly in *Symbiodinium*, is believed to be proteobacterial in origin and most likely acquired through evolutionary lateral gene transfer between dinoflagellates and associated bacteria [64]. In addition, proteomic analysis revealed the presence of the protein peridinin chlorophyll a-binding protein precursor, usually encoded by the plastid genome (Table S6). This is consistent with the idea that the complex is observed among organisms that use rubisco II [65]. This complex is normally observed among photosynthetic dinoflagellates such as *Symbiodinium*. In contrast, dinoflagellates that do not have peridinin containing plastids such as the *Gymbodiniale* lineages, and *Dinophysis* spp. possess rubisco form I instead of the rubisco II [66].

*P. bahamense* is a bioluminescent organism that emits blue light along the coasts of Puerto Rico, the Caribbean islands, Mexico, and Florida [67,68,69]. *P. bahamense* exhibited similar total stimulable light in both the natural and laboratory conditions, with 2.8 × 10^8^ ± 15% (mean photons per organism) during the dark period [70,71]. Bioluminescent proteins such as luciferin-binding proteins were observed in the present study (Table S6). The cDNA library of *Alexandrium catenella* showed luciferin-binding protein as one of the top genes dominating the library, accounting for ~15.6% [72]. Bioluminescence occurs as a chemical reaction between luciferin, luciferase, and oxygen in the presence of salt to produce deoxyluciferin, light, and water. It is often triggered by a drop in pH due to an influx of protons within the cell [73,74,75].

#### 2.1.5. Circadian Rhythm and Growth

Proteins among dinoflagellates that follow the circadian rhythm include those involved in glycolysis and the Krebs cycle [76,77]. Several proteins involved in glycolysis and the Krebs cycle were identified in this study, such as citrate synthase, pyruvate kinase, glyceraldehyde dehydrogenase, 2-oxoglutarate dehydrogenase, succinyl-CoA-3-ketoacid CoA transferase, and tricarboxylate transporter (Table S7). In *Lingulodinium polyedrum*, circadian-regulated proteins are synthesized and degraded daily as a mechanism to conserve nitrogen. Amino acids are generated from the hydrolysis of one protein and consequently, can be made available for the synthesis of different proteins during the circadian cycle [74].

Osmotic growth proteins are expressed by *P. bahamense* and aid in the growth regulation of *P. bahamense* across different salinity conditions. These proteins facilitate the ability of *P. bahamense* to adapt to varying water flux outside and inside the cell and corroborate the observed ability of *P. bahamanse* to grow under a wide range of salinities (10–36 psu) [7].

### 2.2. Characterization of Lipids of P. bahamense

Five percent of the proteins identified using shotgun proteomics are involved in lipid metabolism (Figure 2) in *P. bahamense*. The identified proteins include enoyl-[acyl-carrier-protein] reductase [NADH], 3-oxoacyl-[acyl-carrier-protein] reductase, 3-hydroxyacyl-[acyl-carrier-protein] dehydratase FabA, and biotin carboxylase.

**Figure 6:**
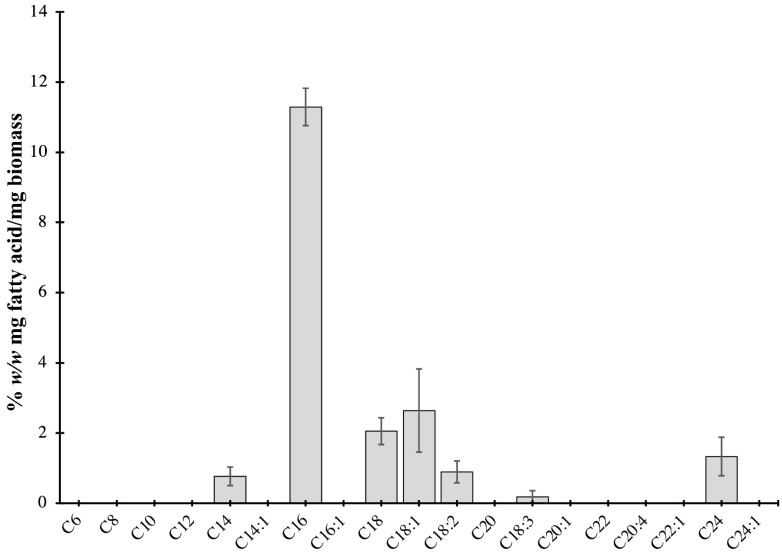
Fatty acid content of crude lipid extracts of *P. bahamense.* Saturated fatty acids (83.57% of crude lipid extract) were the major fatty acids in *P. bahamense*. These include palmitic acid (C16, 62.7%) and stearic acid (C18, 10.3%). Other fatty acids in *P. bahamense* include monounsaturated fatty acids (11.67%) such as oleic acid (C18:1, 14.3%) and polyunsaturated fatty acids (4.76%). Lipids were extracted using the Bligh–Dyer method, converted to the corresponding fatty acid methyl esters, and analyzed by gas chromatography–flame ionization detector (GC-FID). Data are shown as mean ± SD of two biological replicates with three technical replicates each.

To identify and quantify the lipids in *P. bahamense*, extracted lipids were converted to the corresponding fatty acid methyl esters prior to gas chromatography analyses. The predominant fatty acid in *P. bahamense* is palmitic acid (C16, 62.7%), followed by oleic (C18:1, 14.3%) and stearic acid (C18, 10.3%) (Figure 6). The majority of the detected fatty acids are saturated (SFA), accounting for 83.57% of the crude lipid extracts. Monounsaturated fatty acids (MUFA) and polyunsaturated fatty acids (PUFA) account for 11.67% and 4.76%, respectively (Figure 6).

Different fatty acids in dinoflagellates have been used as bioindicators of various cellular behaviors and ecological responses. The ratio of the amount of polyunsaturated fatty acids (PUFA) to saturated fatty acids (SFA) can distinguish benthic from planktonic dinoflagellates [78]. Consistent with the observation, the vegetative form of the planktonic dinoflagellate *P. bahamense* has a PUFA:SFA ratio of 0.057. In contrast to cold-adapted dinoflagellates, *P. bahamense* has low levels of unsaturated fatty acids, presumably due to its adaptation to tropical environments. An increased abundance of unsaturated fatty acids is essential to maintain membrane fluidity [79] and is relevant to phytoplankton in temperate regions [80]. Docosahexaenoic acid (DHA) and eicosapentaenoic acid (EPA) were previously suggested to be possible lipid biomarkers for dinoflagellates [81]. Although DHA and EPA were not quantified in *P. bahamense* because of the lack of standards, C18:1 and C18:3 are known precursors in the production of DHA and EPA in microalgae [82]. Some of the identified fatty acids in *P. bahamense* have also been previously shown to have ecological significance in other dinoflagellate species. PUFAs, in particular, are involved in algal dominance and inhibitory to zooplankton and other animals [83]. PUFA and reactive oxygen species (ROS) produced by *Alexandrium* species have been reported to cause synergistic reactions, which can cause gill damage in fish [84]. In our findings, *P. bahamense* mostly produces C16, C18, and C18:1 as the major fatty acids. These fatty acids, together with linoleic acid (octadecadienoic, C18:2), and linolenic acid (octadecatrienoic, C18:3), are the most abundant fatty acids found in microalgae [85]. The production of these fatty acids may play a role in the ability of *P. bahamense* to inhibit the growth of other algal species and zooplankton in the environment during *P. bahamense* bloom.

### 2.3. Carbohydrate Content of P. bahamense

Shotgun proteomics analysis revealed that most proteins in *P. bahamense* are involved in carbohydrate degradation (22%), carbohydrate metabolism (18%), and carbohydrate biosynthesis (2%) (Figure 2). Carbohydrate biosynthesis in *P. bahamense* was mapped onto the gluconeogenesis pathway. On the other hand, carbohydrate degradation in *P. bahamense* is mainly carried out through the glycolysis and pentose phosphate pathway.

Carbohydrate metabolism through the TCA cycle in *P. bahamense* was mapped in three steps: (a) fumarate from succinate (bacterial route), (b) isocitrate from oxaloacetate, and (c) succinate from succinyl-CoA ligase (ligase route). The TCA cycle is a series of chemical reactions that are performed by aerobic organisms to release stored energy through the oxidation of acetyl-CoA from carbohydrates, fats, and proteins. Final products include ATP production and carbon dioxide [86]. Genes involved in carbohydrate transport and metabolism, in particular those involved in the TCA cycle, glycolysis/gluconeogenesis, and pentose phosphate, were previously shown to be highly expressed in bloom communities [87]. These pathways enable energy generation for the dinoflagellate [87,88]. In the case of *Symbiodinium*, glucose was identified as the major transferred metabolite between dinoflagellate–cnidarian symbiosis [89]. Through glycolysis and gluconeogenesis, dinoflagellates can regulate glucose homeostasis in the cell [90].

**Figure 7:**
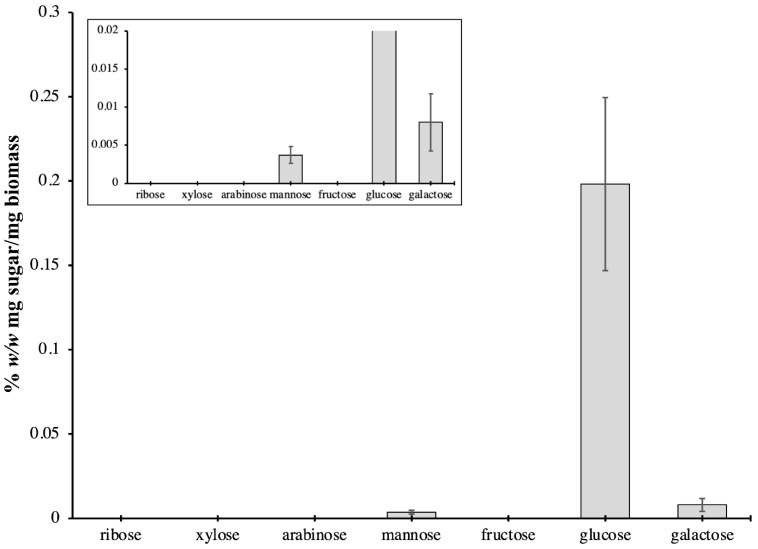
Carbohydrate content of *P. bahamense*. Sugar profiling of *P. bahamense* showed glucose as the major constituent (93.0%). Other sugars comprised 7.0% of the total carbohydrate content. Mannose and galactose concentrations were 0.0037% and 0.0080% *w*/*w* (mg sugar/mg biomass), respectively. Carbohydrates were extracted from *P. bahamense* biomass using the phenol–sulfuric acid method, and the corresponding monosaccharides were analyzed using HPLC-MS with multiple reaction monitoring (MRM) detection. Data are presented as mean ± SD of two biological replicates with three technical replicates each.

Glucose was detected as the main sugar component of *P. bahamense*, comprising 93.0% of the total sugars analyzed (Figure 7). Mannose and galactose concentrations were 0.0037% *w*/*w* (mg sugar/mg biomass) and 0.0080% *w*/*w* (mg sugar/mg biomass), respectively (Figure 7).

The dominance of glucose is due to cellulose being the main component of the cell wall [91] and amphiesma [92] of dinoflagellates. The presence of mannose in *P. bahamense* may be attributed to the presence of non-cellulosic β-glucans such as mannan. These carbohydrates are more common and well documented in plants and used as storage polysaccharides. However, mannan has been also documented in algae, particularly in green algae [93,94]. Mannose is the main sugar in the diatom *Phaeodactylum tricornutum*, which is either derived from a structural mannan in the cell wall or associated with glycoproteins in the cell matrix [95]. It is possible that some non-cellulosic β-glucans, such as hemicellulose, also constitute the cell wall of *P. bahamense*. The main structural component of the cell wall of the dinoflagellate *Peridinium westii* is a polysaccharide that is formed from glucose subunits but structurally different from cellulose [96]. Galactose was possibly derived from complex galactans ramified by glucose, which is also observed in different algal species [97]. In *Chlorella vulgaris*, the major sugars are glucose and galactose, which are present in almost equal amounts, at 30% of the total sugars [95]. Free galactose may be derived in large concentrations of galactolipids (e.g., MGDG, DGDG), which make up the photosynthetic membranes in actively growing cells, and β-(1-6)-linked galactans decorate the cell wall glycoproteins [95]. The possible presence of these polysaccharides may explain the rigidity of the vegetative cell wall and resistant cyst wall of *P. bahamense*. Further, the life strategy of *P. bahamense*, which consists of producing highly resistant cysts capable of obligate dormancy and temporary thin-walled cysts that can easily be converted to vegetative cells, make this organism eco-physiologically important [16]. Our biochemical analyses signify that *P. bahamense* may be producing different cell wall materials as needed. Further characterization of these specific saccharides will aid in the understanding of the cell wall dynamics in *P. bahamense*.

## 3. Materials and Methods

### 3.1. Cultivation of P. bahamense

Cultures of *Pyrodinium* *bahamense* (code: *PBCMZRVA042595*) were obtained from the Red Tide Laboratory of Prof. Rhodora V. Azanza from the Marine Science Institute, University of the Philippines. This culture was collected from Masinloc Bay, Zambales, Philippines on 25 April 1995. The morphological and genomic identification of the culture was previously reported by Gedaria et al. (2007) [7]. Subculturing of monoclonal *P. bahamense* using F/2 culture medium was performed with the following parameters: temperature of 24 °C (±2), 12:12 h light:dark cycle, light intensity of 200 ± 50 μEm^−2^s^−1^ [31,32,98]. Manual cell counts using a Sedgewick rafter counting chamber were taken every 4–5 days to monitor growth. Starting cell density was 200 cells/mL. Two biological replicates were conducted. Cells for proteomics were harvested at the exponential phase during the light period. The cells were collected by centrifugation for 20–30 min, 4 °C at 500× *g* (2×). Cells were washed with sterile filtered seawater and stored at −80 °C until further use.

### 3.2. Moisture and Ash Content

Residual moisture and ash content of freeze-dried biomass of *P. bahamense* were determined according to AOAC Official Method 930.15 [99] and AOAC Official Method 942.05 [100], respectively.

### 3.3. Lipid Extraction and Analysis

Lipid extraction was done according to the method of Bligh and Dyer (1959) [101]. A total of 2 L of culture of *P. bahamense* was centrifuged at 1200× *g* for 10 min. The cell pellet (100 mg) was lyophilized and extracted with 1:2 (*v*/*v*) CHCl_3_:CH_3_OH. The organic extract was collected and dried under reduced pressure to yield the total lipid extract. Lipids were converted to the corresponding fatty acid methyl esters (FAMEs) using the AOAC official method 969.33 [102]. Identification and quantitation of FAMEs were performed using gas chromatography with flame ionization detector (Shimadzu, Kyoto, Japan) by comparing with available standards using the AOAC Official Method 963.22 [103]. Analysis was performed in two biological replicates with three technical replicates each. Results are presented as % *w*/*w* (mg fatty acid/mg crude lipid extract).

### 3.4. Carbohydrate Extraction and Analysis

The carbohydrate content of *P. bahamense* biomass was extracted using the method of Templeton et al. (2012) [95]. Lyophilized biomass (25 mg) was extracted with 72% (*w*/*w*) H_2_SO_4_ at 30 °C for 1 h. The reaction was terminated by adding H_2_O to bring the H_2_SO_4_ concentration to 4% (*w*/*w*), autoclaved and filtered using a Phenex-RC 0.2-μm syringe filter (Phenomenex, Torrance, CA, USA). Total concentration of monosaccharides, disaccharides, and polysaccharides was determined using the phenol–sulfuric acid colorimetric method [104]. Sugar analysis was performed using the method of Schulze et al. (2017) [35] with modifications. The following instrument setup was used for analysis: Shimadzu LC-20AD liquid chromatograph with SIL-20AHT autosampler. Chromatographic separation was performed on an Acquity UPLC BEH Amide column 1.7 µm, 2.1 × 50 mm (Waters, Milford, MA, USA). A total of 1 µL aliquot of each sample was eluted at 0.2 mL/min by a gradient program of CH_3_CN/5 mM NH_4_HCO_2_ (both with 0.1% formic acid modifier), 90–75% CH_3_CN in 8.5 min, and 75% CH_3_CN for 4 min. Detection was carried out by multiple reaction monitoring (MRM) analysis (Shimadzu LCMS-8040, Shimadzu, Kyoto, Japan). The optimized transitions for each standard are shown in Table S2. Data were analyzed with LabSolutions (Shimadzu) using manual peak integration. External calibration curves for each standard were prepared by plotting the integrated peak area vs. the concentration. Five concentrations (0.156–0.0098 µg/mL) in two-fold serial dilutions were prepared and injected three times for repeatability. Results are presented as % *w*/*w* (mg sugar/mg biomass).

### 3.5. Proteome Analysis

#### 3.5.1. Protein Extraction and Quantitation

A total of 2.0 × 10^6^ cells was used per biological replicate for protein extraction. Proteins were extracted using a modified urea triton X-100 buffer with TCA/acetone precipitation [31] in the presence of a protease inhibitor cocktail (AEBSF—[4-(2-Aminoethyl)benzenesulfonyl fluoride hydrochloride], aprotinin, bestatin hydrochloride, leupeptin hemisulfate salt, pepstatin A, and E-64—[N-(trans-Epoxysuccinyl)-L-leucine 4-guanidinobutylamide]) (1% *v*/*v*). The cell pellet was lysed on ice using an ultrasonic probe (Cole Parmer, Vernon Hills, IL, USA) at 60 Hz for 3 min in 5 s bursts. Samples were centrifuged at 15,000× *g* for 30 min at 4 °C. The supernatant was collected, and proteins were precipitated by adding 20% TCA/acetone solution (*w*/*v*) at 4 °C for 30 min. The protein pellet was collected by centrifugation at 15,000× *g* for 30 min at 4 °C, washed with cold acetone containing 20 mM DTT, and subsequently air-dried to remove residual acetone. Two biological replicates were performed.

#### 3.5.2. Mass Spectrometry Analysis

Mass spectrometric analysis was performed by Proteome Factory (Proteome Factory AG, Berlin, Germany). Protein pellets were dissolved in a reducing dissolution buffer (6 M of urea, 100 of mM tris/HCl pH of 8.3). Bradford assay was used to quantify the dissolved pellets. The samples were reduced, alkylated, and digested in solution. In brief, DTT was added to a final concentration of 5 mM to the dissolved protein sample and incubated for 25–45 min at 56 °C to reduce the disulfide bonds. Iodoacetamide was added to a final concentration of 14 mM. The reaction was quenched after 30 min with the addition of DTT. Proteins were digested using trypsin (4–5 ng/µL), and the reaction was terminated after 24 h with the addition of TFA (0.4% *v*/*v*). The peptide solution was trapped and desalted using an enrichment column (Zorbax SB C18, 0.3 mm × 5 mm, Agilent, Santa Clara, CA, USA) for 5 min with an isocratic elution of 99.5% CH_3_CN/0.5% aqueous HCOOH as eluent at a flow rate of 25 µL/min.

Peptides were analyzed using reversed phase chromatography on an Agilent HPLC system coupled with an LTQ Orbitrap XL (Thermo Fisher, Waltham, MA, USA) mass spectrometer. The chromatographic conditions were as follows: Zorbax 300 SB C18, 5 μm, 75 mm × 150 mm (Agilent) and CH_3_CN/0.1% aqueous HCOOH as mobile phase with a flow rate of 350 nL/min, using a gradient of 10–32% CH_3_CN in 45 min (60 min gradient) or from 2–20% CH_3_CN in 90 min and further to 32% CH_3_CN in 13 min (120 min gradient). The mass spectrometry (MS) system consisted of an Agilent 1100 nanoLC system (Agilent, Waldbronn, Germany), PicoTip electrospray emitter (New Objective, Woburn, MA, USA), and an Orbitrap XL mass spectrometer (Thermo Fisher, Bremen, Germany).

#### 3.5.3. Protein Identification and Annotation

Mass spectrometric data were analyzed using MASCOT v2.5.1 (Matrix Science: Ohio, USA, 2014) unrestricted (30 September 2016) and restricted MaxQuant v1.5.5.1 (Max Planck Institute of Biochemistry: Munich, Germany, 2016) database searches (30 September 2016). MaxQuant peptide identification was performed against the available NCBInr databases for *Ruegeria atlantica*/*Marinovum algicola*, *Cylindrospermopsis raciborskii*, and *Alexandrium* sp. Protein identification utilized the available mRNA database for *Symbiodinium* *kawagutii* Trench and Blank, 2000 (http://web.malab.cn/symka_new/; accessed on 1 September 2016) maintained by Prof. Quan Zhou’s group at Xiamen University [39], and in-house transcriptome data for *P. bahamense*. *P. bahamense* mRNA information is readily available at NCBI accession: PRJNA261863, ID: 261863. The mRNA sequences were translated into protein sequences using transeq from the EMBOSS Explorer [105]. The output file was used for subsequent MaxQuant analysis for protein identification.

For both MASCOT and MaxQuant analysis, standard settings were used. Deamidation of asparagine and glutamine and methionine oxidation were set as variable modification, while carbamidomethylation was set as fixed modification. Threshold parameters for the query were as follows: peptide mass tolerance: ±5 ppm, fragment mass tolerance: ±0.6 Da, maximum missed cleavage: 2, mass values: monoisotopic.

Identified hits were filtered from protein hits that were “only identified by site” (proteins only identified by a modification site), “reverse hits” (protein hits with at least 50% of the peptides derived from the reversed part of the decoy database) and “contaminants” (protein hits from keratin and auto-lysis of trypsin). Proteins were identified using reference databases based on unique peptides with a standard setting of seven amino acids as minimum peptide length. Protein identities were then determined using the protein score, sequence coverage, and q-value as parameters [106,107].

After filtering potential contaminants, proteins were identified using at least one unique peptide with seven amino acids as minimum peptide length. Peptide-to-spectrum (PSM), false discovery rate (FDR), protein FDR, and site FDR were set at 1% (0.01). Protein identification and search score were set to >0 for unmodified peptides and >40 for modified peptides [108,109]. For exploratory and baseline proteome information using shotgun proteomics, no protein score threshold was set [110,111]. Protein identification was made using the FDR strategy and *q*-value threshold strategy, in which the top-ranking peptide identification satisfying an FDR < 1% and *q*-value set at <0.1 (95% confidence interval) were used [110,112,113].

The *q*-value, which is the adjusted statistical *p*-value with respect to the FDR, was used in this study as an indication of the level of false measurements. The *q*-value of a statistic *t* defined as the minimum FDR incurred by declaring *t* significant. FDR is defined as the proportion of all significant tests that are expected to be false and is estimated as the *E*-value divided by the number of predictions made. The *q*-value and FDR do not increase with the database size N, unlike the *E*-value; hence, predictions do not usually lose significance with the FDR as the database grows. *Q*-values range lie between 0–1, with 0 as the best score. A cut-off q-value score of <0.1 was adapted for this study. Overall, *q*-values were proven to statically outperform *E*-values as measures of significance [114]. Moreover, the *q*-value is regarded as the best method of choice for protein false discovery rate estimation in large proteomic data sets such as in shotgun proteomics [113].

Quantitation of peptides was performed using label-free quantitation (LFQ) intensities—spectral counting of peptides unique to a specific protein. LFQ intensities were log2-transformed and normalized by dividing the median LFQ intensity separately for each sample.

LFQ=(protein signal intensity)/(median signal intensity)

Annotations were performed using UniProt (https://www.uniprot.org/; accessed on 1 December 2016), Gene Ontology (GO, https://www.uniprot.org/uploadlists/; accessed on 1 December 2016), Reduced and Visualized Gene Ontology (REVIGO, http://revigo.irb.hr/; accessed on 30 June 2021), and Kyoto Encyclopedia of Genes and Genomes (KEGG, https://www.genome.jp/kegg/kegg_ja.html; accessed on 1 December 2016) analysis.

## 4. Conclusions

Characterization of the proteins, carbohydrates, and lipids of *P. bahamense* based on mass spectrometry and chromatographic analyses provided baseline information on the biochemical characteristics of *P. bahamense* at the vegetative stage. These gave new insights into the cellular machinery behind this environmentally important organism. The molecular characterization has, in part, corroborated previous observations on the biological and ecological behaviors of *P. bahamense*. The biochemical analyses further highlighted the contribution of microorganisms associated with *P. bahamense*.

## Data Availability

Mass spectrometry and proteomics data are deposited at the Japan Proteome Standard Repository/Database (jPOST): JPST001415/PXD030176.

## Supplementary Materials

The following are available online at https://www.mdpi.com/article/10.3390/ijms222413332/s1.
