# Supplementary material for: Biochemical Mapping of Pyrodinium bahamense Unveils Molecular Underpinnings behind Organismal Processes
Source: Int J Mol Sci. 2021 Dec 11;22(24):13332. doi: 10.3390/ijms222413332 (PMC8706660; doi:10.3390/ijms222413332)
Supplement: Supplementary file 1 [file ijms-22-13332-s001.zip › SI_Salvador-Reyes_Pyrodinium_revised1_12012021.pdf]

In-depth biochemical mapping of *Pyrodinium bahamense* highlights the contribution of associated microorganisms to critical cellular processes

Bryan John J. Subong[1,2], Zabrina Bernice L. Malto[1], Arturo O. Lluisma[1],
Rhodora V. Azanza[1], Lilibeth A. Salvador-Reyes[1*]

[1] Marine Science Institute, University of the Philippines- Diliman, Velasquez St., UP Diliman, Quezon City, 1101 Philippines
[2] Present Address: Department of Chemistry, The University of Tokyo, 7-3-1 Hongo, Bunkyo City, Tokyo 113-8654 Japan

*corresponding author: lsreyes@msi.upd.edu.ph

Supplemental Figures

Figure S1. Biomass composition of *P. bahamense*.
Figure S2. Summary of unique and similar GeneInfo identifier (GI) numbers for the two biological replicates of *P. bahamense*.
Figure S3. Protein distribution based on taxonomy (A), protein ontology (B) and enzyme class (C).
Figure S4. Protein distribution based on molecular function (A), cellular component (B) and biological process (C).
Figure S5. REVIGO analysis of combined GO identifiers of proteins identified using various reference databases.
Figure S6. Toxin content of *P. bahamense.*

Supplemental Tables

Table S1. Optimized MRM Transitions for Monosaccharide Standards
Table S2. Proteins from Potential Associated Bacterial Interactions
Table S3. Proteins involved in Toxin Biosynthesis
Table S4. Proteins involved in Arginine Biosynthesis
Table S5. Polyketide Synthesis-related Proteins
Table S6. Proteins Involved in Photosynthesis and Bioluminescence
Table S7. Proteins Involved in Circadian Rhythm and Growth

Supplemental Datasets

Data S1. MASCOT_NCBInr database search
The file contains a tabulated protein identification and annotation using MASCOT NCBInr database search.

Data S2. Protein identification across five reference taxa
The file contains a tabulated protein identification and annotation using various reference proteomes/transcriptomes of *Marinovum algicola*, *Alexandrium*, *Cylindrospermopsis raciborskii*, *Symbiodinium kawagutii* and *Pyrodinium bahamense*.

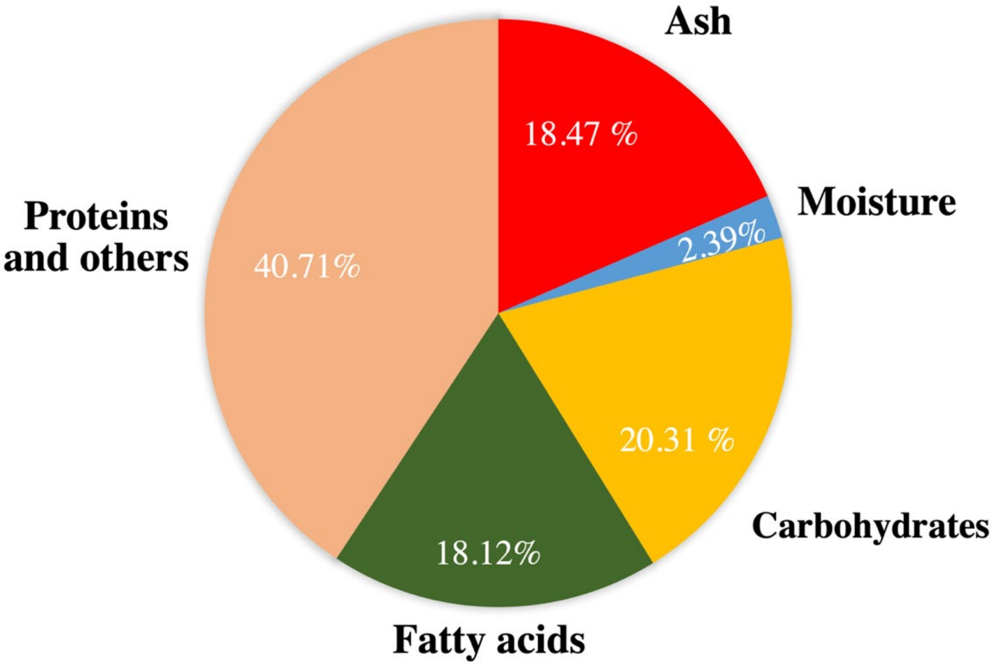
Ash
18.47 %
Moisture
2.39%
Proteins
and others
40.71%
20.31 %
Carbohydrates
18.12%
Fatty acids

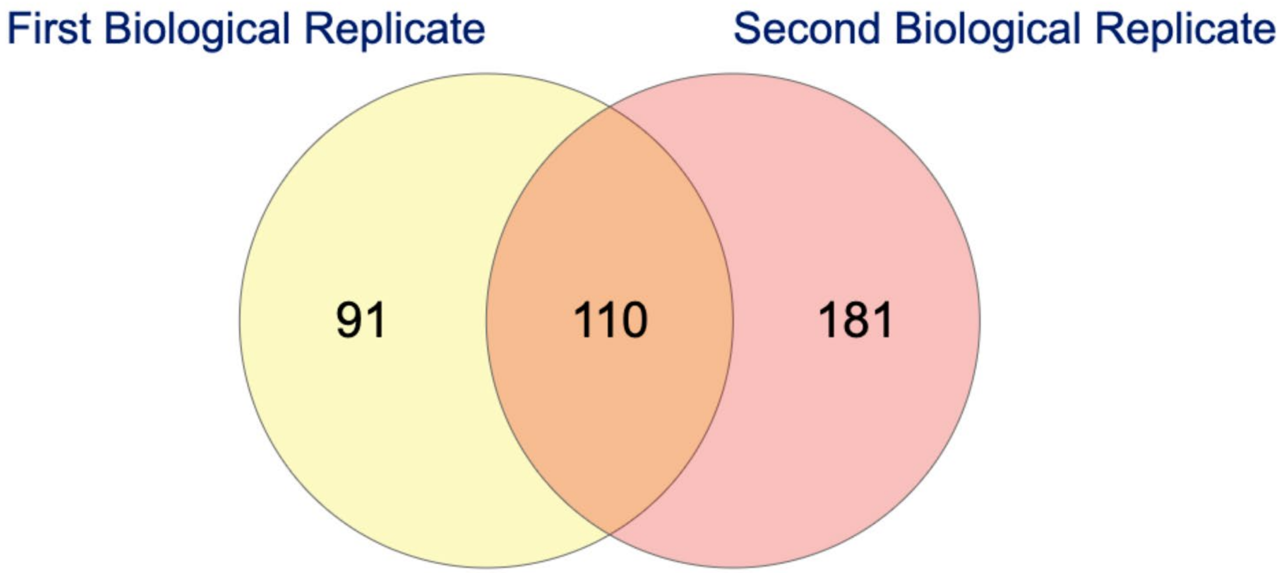

Figure S2. Summary of unique and similar GeneInfo identifier (GI) numbers for the two biological replicates of *P. bahamense*. 110 GI were common for both replicates while there were 91 and 181 unique GI numbers for the first and second biological replicate, respectively.

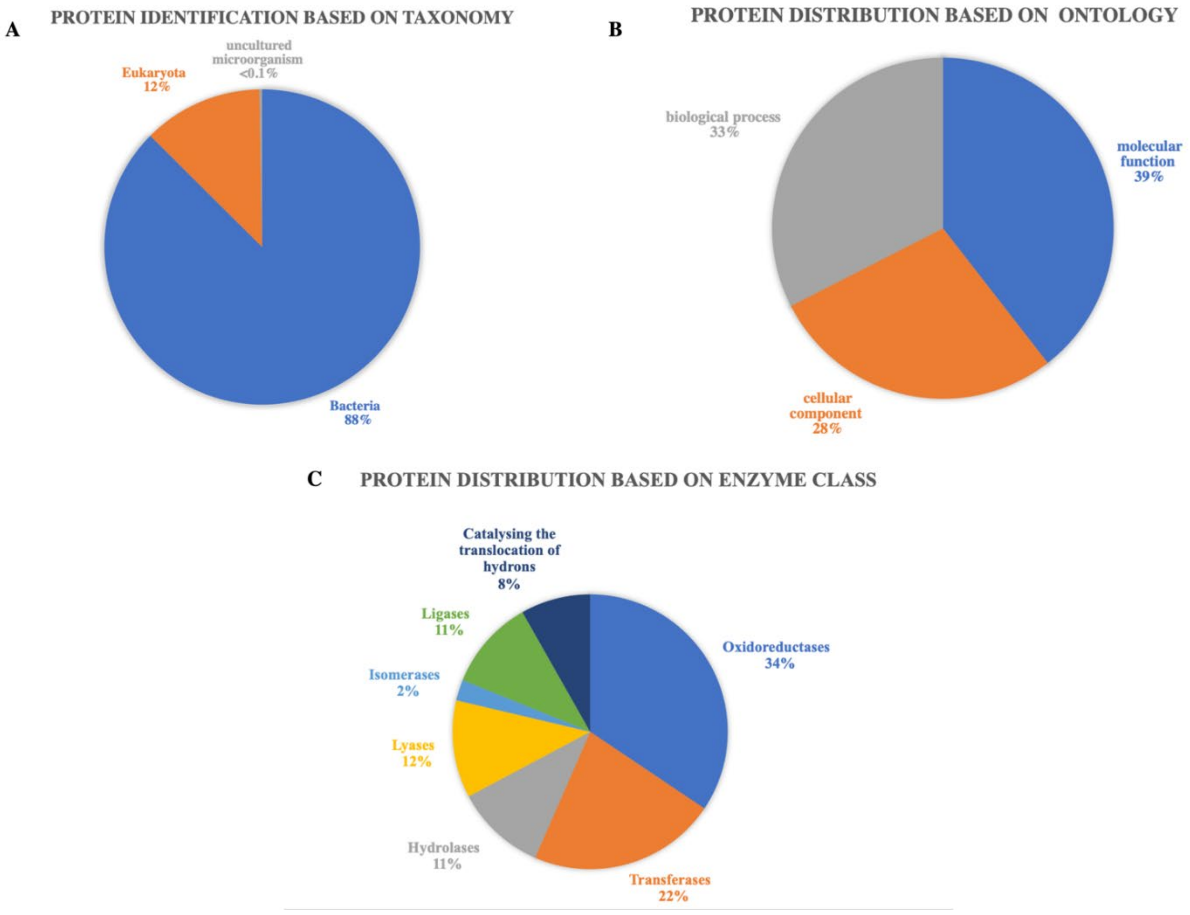
A
PROTEIN IDENTIFICATION BASED ON TAXONOMY
uncultured microorganism <0.1%
Eukaryota 12%
Bacteria 88%
B
PROTEIN DISTRIBUTION BASED ON ONTOLOGY
biological process 33%
molecular function 39%
cellular component 28%
C
PROTEIN DISTRIBUTION BASED ON ENZYME CLASS
Catalysing the translocation of hydrons 8%
Ligases 11%
Isomerases 2%
Lyases 12%
Hydrolases 11%
Transferases 22%
Oxidoreductases 34%

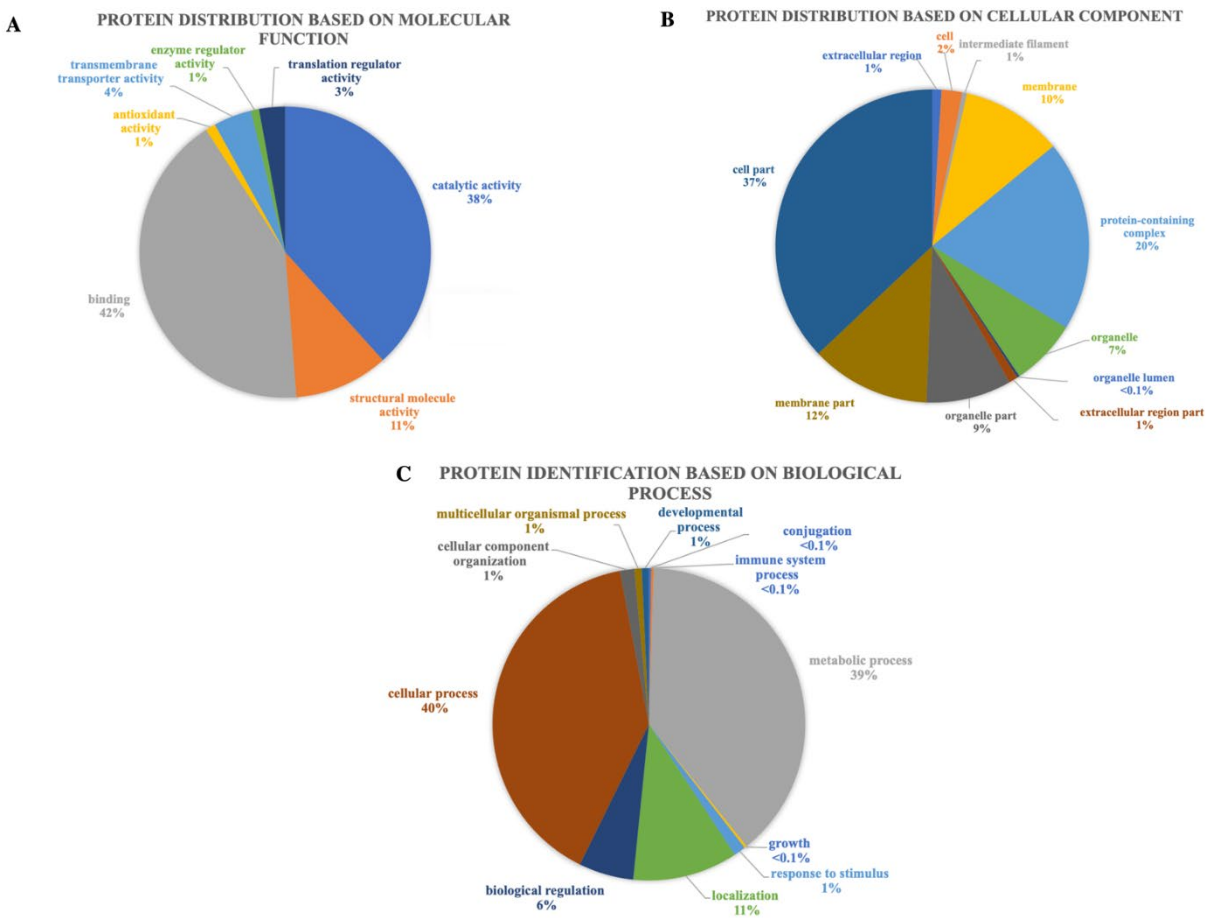
A
PROTEIN DISTRIBUTION BASED ON MOLECULAR FUNCTION
enzyme regulator activity 1%
transmembrane transporter activity 4%
translation regulator activity 3%
antioxidant activity 1%
catalytic activity 38%
binding 42%
structural molecule activity 11%
B
PROTEIN DISTRIBUTION BASED ON CELLULAR COMPONENT
extracellular region 1%
cell 2%
intermediate filament 1%
membrane 10%
cell part 37%
protein-containing complex 20%
organelle 7%
organelle lumen <0.1%
membrane part 12%
organelle part 9%
extracellular region part 1%
C
PROTEIN IDENTIFICATION BASED ON BIOLOGICAL PROCESS
multicellular organismal process 1%
developmental process 1%
conjugation <0.1%
cellular component organization 1%
immune system process <0.1%
metabolic process 39%
cellular process 40%
growth <0.1%
response to stimulus 1%
biological regulation 6%
localization 11%

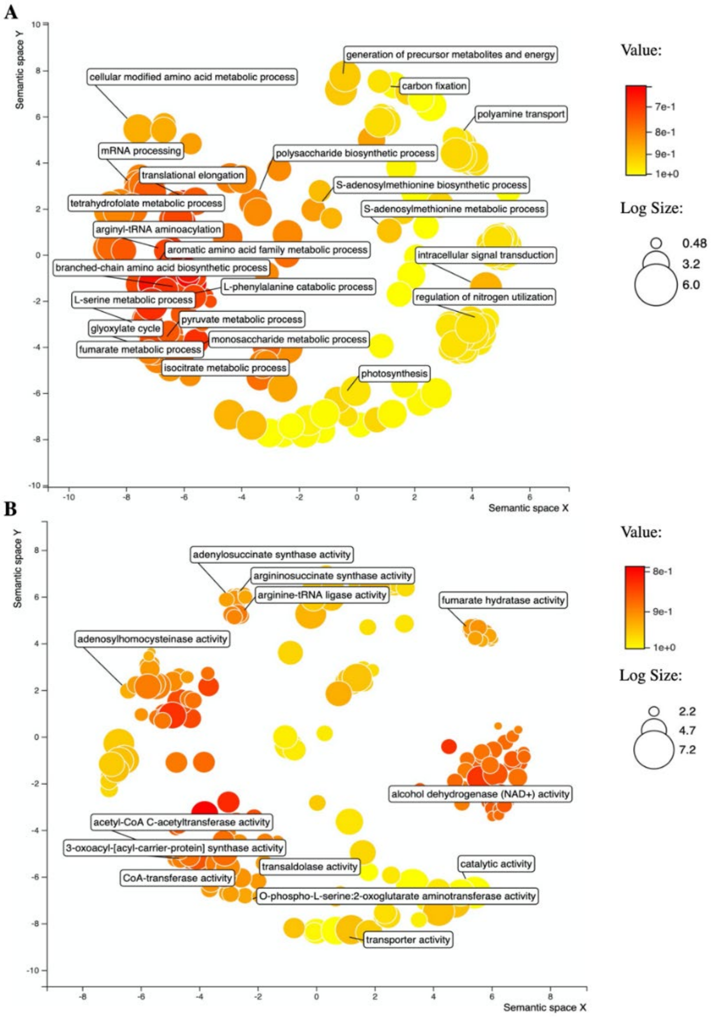

Figure S5. REVIGO analysis of combined GO identifiers of proteins identified using various reference databases. The GO identifiers obtained from proteins identified across different reference databases were visualized using REVIGO to map the various processes for *Pyrodinium bahamense*. **A**.) Notable biological processes include amino acid biosynthesis and degradation, polysaccharide biosynthesis, S-adenosylmethionine biosynthesis and metabolism, glycolysis, and photosynthesis. **B.**) Meanwhile, important molecular functions include adenosylsuccinate synthase and adenosyl homocysteinase activity, glycolysis-related activities, catalysis and transporter activities. Bubble color indicates the p-values for the false discovery rates while the circle log size indicates the logarithmic frequency of the GO term.

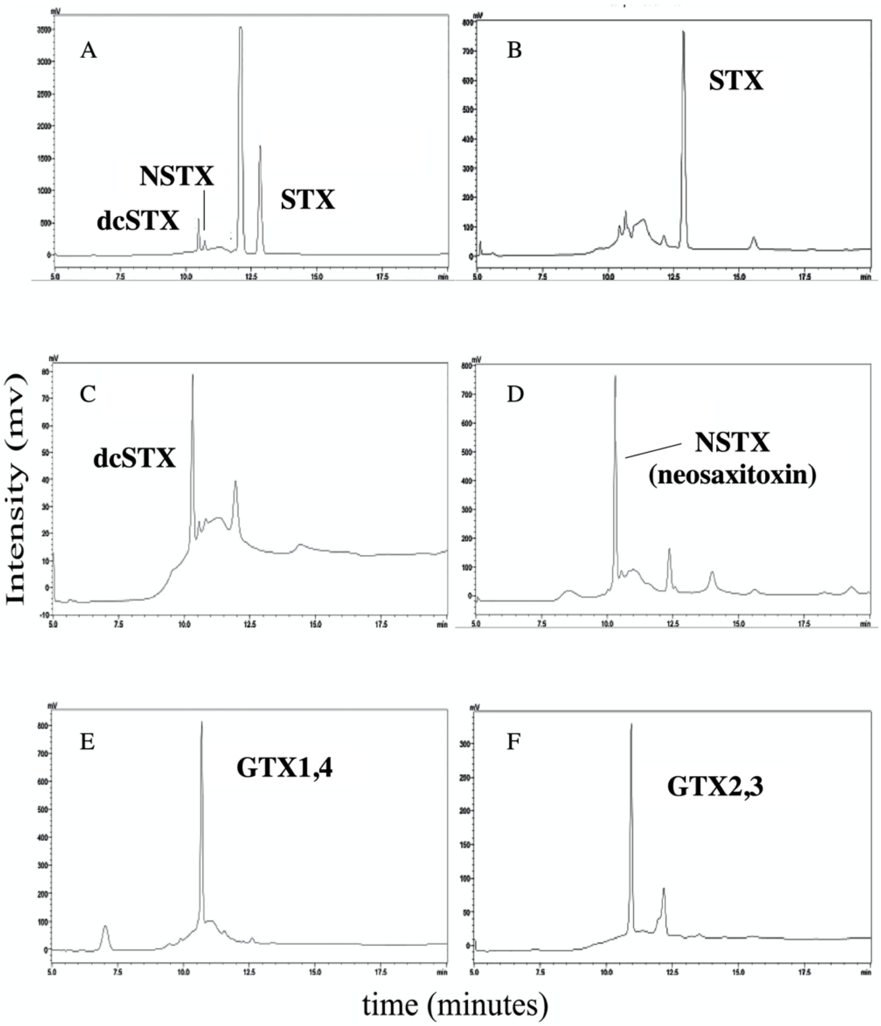

Figure S6. Toxin content of *P. bahamense*. A. A pre-column oxidation method for toxin content using available toxin standards for quantification was conducted based on the procedures of Subong, et al. 2017 and Subong, et al. 2021. Subcltured *P. bahamense* used for proteomic analysis showed toxin content of 200-215 fmol STX equiv/cell with toxin profiles consist of STX, neoSTX and dcSTX (A). Toxin content and profile was compared to standard **B**. saxitoxin (STX); **C**. decarbamoylsaxitoxin; **D**. neosaxitoxin (NSTX); **E**. gonyautoxin 1,4; **F**. gonyautoxin 2,3.

Supplementary Tables

Table S1. Optimized MRM Transitions for Monosaccharide Standards

Standard	Transition	Product m/z	CE
Arabinose	194.90 > 89.05	89.05	11
	194.90 > 149.20	149.20	9
	194.90 > 59.05	59.05	20
Fructose	224.95 > 89.05	89.05	13
	224.95 > 179.15	179.15	6
	224.95 > 59.10	59.10	23
Galactose	224.90 > 89.05	89.05	12
	224.90 > 179.15	179.15	7
	224.90 > 59.10	59.10	22
Glucose	224.95 > 179.15	179.15	7
	224.95 > 89.05	89.05	12
	224.95 > 59.05	59.05	23
Mannose	224.95 > 179.15	179.15	7
	224.95 > 119.10	119.10	10
	224.95 > 89.10	89.10	13
Ribose	194.90 > 149.20	149.20	8
	194.90 > 89.05	89.05	12
	194.90 > 59.10	59.10	20
Xylose	194.90 > 89.05	89.05	11
	194.90 > 149.20	149.20	9
	194.90 > 59.10	59.10	17

Table S2. Proteins from Potential Associated Bacterial Interactions

Protein	Number of peptides	Sequence Coverage (%)	Score	q-value	Function
amino acid ABC transporter-substrate binding proteins**<br><br>*Sequence: NTTWTFSRDTSMGLYF*<br><br>[*Cylindrospermopsis raciborskii*]	1	2.3	132	0	solute active transporter
elongation Tu factor<br><br>[*Alexandrium*]	10	32.5	262	0	catalysts for aminoacyl-tRNA (aa-tRNA) binding to the ribosome
RHS-repeat associated core-containing protein<br><br>[*Marinovum algicola*]	67	38.1	323	0	bacterial exotoxin
bifunctional folylpolyglutamate synthase/dihydrofolate synthase**<br><br>*Sequence: VGAGQMSRVDSALIAA*<br><br>[*Marinovum algicola*]	1	2.9	1.6*	0	synthesis of folate
propionyl-CoA synthethase**<br><br>*Sequence: TFAELQTRVATLAGAL*<br><br>[*Marinovum algicola*]	1	8.7	1.2*	0.007	propanoate metabolism

acryloyl-CoA reductase [*Marinovum algicola*]	2	7.9	0.98*	0.007	L-alanine fermentation

** denotes MaxQuant Score; otherwise, MASCOT score is reported*
*** unique peptide*

Table S3. Proteins involved in Toxin Biosynthesis

Protein	Number of peptides	Sequence Coverage (%)	Score	q-value	Function
chlorophyll a/c- binding protein, partial<br><br>(*Alexandrium tamarense)*	2	9.1	22*	0	photosynthesis
RHS repeat-associated core domain-containing protein<br><br>[*Marinovum algicola*]	67	38.1	323	0	bacterial exotoxin
contig07438 adenosylhomocysteinase<br><br>[Pbc transcript]	3	16.4	2.5*	0	methylation
methyltetrahydropteroyltriglutamate-homocysteine S-methyltransferase activity**<br><br>*Sequence:*<br>*GLITLNPKREAVSSLA*<br>[*Symbiodinium kawagutii* transcript]	1	0.7	5.8*	0.0175	L-methionine biosynthesis
acetyl-CoA acetyltransferase<br><br>[*Alexandrium*]	2	15	17*	0	transferase activity
contig00523 methionine S-adenosyl transferase [Pbc transcript]	2	6.9	5.00*	0	cysteine and methionine metabolism
periplasmic sensor hybrid histidine kinase **<br><br>*Sequence:*<br>*GDLMVVSRPGHGATFF*<br>[*Alexandrium/ Mameliella alba*]	1	2.5	5.9*	0	integral membrane component

iron-containing alcohol dehydrogenase **<br><br>*Sequence:*<br>*TDVKMMMKHPSFTPQV*<br>[*Alexandrium/ Bacillus sp.*]	1	2.2	6.2*	0	redox reaction
contig06470 ferredoxin-binding protein II<br>[Pbc transcript]	8	42	125*	0	photosynthesis
ferredoxin-nitrite reductase **<br><br>*Sequence:*<br>*IPLNVWVKPEEVVSLS*<br>[*Cylindrospermopsis raciborskii*]	1	2.5	6.0*	0	nitrogen metabolism

** denotes MaxQuant Score; MASCOT score if not indicated*
*** unique peptide*

Table S4. Proteins involved in Arginine Biosynthesis

Protein	Number of peptides	Sequence Coverage (%)	Score	q-value	Function
ornithine decarboxylase [*Marinovum algicola*]	3	4.1	5.3*	0	polyamine synthesis
argininosuccinate synthase [*Marinovum algicola*]	7	21	28*	0	*argininosuccinate* synthesis
ornithine-acyl-ACP acyltransferase**<br><br>*Sequence: LAEDQIDRRRAMLQVP*<br>[*Alexandrium*]	1	6.8	5.8*	0	transferase activity
bifunctional ornithine acetyltransferase/N-acetylglutamate synthase**<br><br>*Sequence: VGAGQMSRVDSALIAA*<br>[*Marinovum algicola*]	1	2.9	1.6*	0	arginine biosynthesis

** denotes MaxQuant Score; otherwise, MASCOT score is reported*
*** unique peptide*

Table S5. Polyketide Synthesis-related Proteins

Protein	Number of peptides	Sequence Coverage (%)	Score	q-value	Function
type I polyketide synthase mixed NRPS/PKS (CyrB) [*Cylindrospermopsis raciborskii*]	2	1.0	12*	0	polyketide synthesis and non-ribosomal peptide synthesis
beta-ketoacyl-ACP reductase [*Marinovum algicola*]	2	13	34*	0	type II Fatty acid biosynthesis
enoyl-[acyl-carrier-protein] reductase [*Marinovum algicola*]	3	12	3.6*	0	type II fatty acid biosynthesis
ornithine-acyl-ACP acyltransferase ** <br>*Sequence:* *LAEDQIDRRRAMLQVP* <br>[*Alexandrium*]	1	6	6.8*	0	ornithine synthesis
beta-ketoacyl-[acyl-carrier-protein] synthase II <br>[*Marinovum algicola*]	9	50	20*	0	fatty acid biosynthesis
3-hydroxyacyl-[acyl-carrier-protein] dehydratase FabA <br>[*Marinovum algicola*]	4	34	3.8*	0	lipid A biosynthesis

** denotes MaxQuant Score; otherwise, MASCOT score is reported*
*** unique peptide*

Table S6. Proteins Involved in Photosynthesis and Bioluminescence

Protein	Number of peptides	Sequence Coverage (%)	Score	q-value	Function
Rubisco [*Symbiodinium*]	6	7.7	130*	0	carbon fixation
peridinin chlorophyll a-binding protein precursor<br><br>[Pbc transcript]	13	47.9	253*	0	photosynthesis
luciferin binding protein**<br><br>*Sequence: VAQETFTKMVQLARKL*<br><br>[Pbc transcript]	1	3.7%	1.0*	0	bioluminescence

** denotes MaxQuant Score; otherwise, MASCOT score is reported*
*** unique peptide*

Table S7. Proteins Involved in Circadian Rhythm and Growth

Protein	Number of peptides	Sequence Coverage (%)	Score	q-value	Function
citrate synthase<br>[*Marinovum algicola*]	10	29.9	57*	0	citric acid cycle
pyruvate kinase<br><br>[*Marinovum algicola*]	2	7.5	2.4*	0	glycolysis
glyceraldehyde dehydrogenase**<br><br>*Sequence:*<br>*PTSTGAAKAVGLVLPE*<br><br>[*Marinovum algicola*]	1	4.3	6.7*	0	glycolysis
2-oxoglutarate dehydrogenase**<br><br>*Sequence:*<br>*VDGSGDVKYHLGASSD*<br>[*Alexandrium*]	1	2.3	7.0*	0	citric acid cycle
succinyl-COA-3-ketoacid COA transferase**<br><br>*Sequence:*<br>*DTGNLVFRKTARNFNP*<br><br>[*Marinovum algicola*]	1	7.7	24*	0	valine, leucine, isoleucine degradation
tricarboxylate transporter**<br><br>*Sequence:*<br>*QGNLSIPRRMCAGLVG*<br><br>[Pbc transcript]	1	9.4%	2.0*	0.005	citrate transporter

** denotes MaxQuant Score; otherwise, MASCOT score is reported; ** unique peptide*

References:

Subong, B.J.J.; Benico, G.A.; Sulit, A.K.L.; Mendoza, C.O.; Cruz, L.J.; Azanza, R. V.; Jimenez, E.C. Toxicity and protein expression of *Alexandrium* species collected in the philippine waters. *Philipp. J. Sci.* **2017**, *146*, 425–436.

Subong, B.J.J.; Lluisma, A.O.; Azanza, R. V.; Salvador-Reyes, L.A. Differentiating two closely related *Alexandrium* species using comparative quantitative proteomics. *Toxins (Basel).* **2020**, *13*, DOI: 10.3390/toxins13010007, doi:10.3390/toxins13010007
